# Cancer: an emergent property of disturbed resource-rich environments? Ecology meets personalized medicine

**DOI:** 10.1111/eva.12232

**Published:** 2015-03-26

**Authors:** Hugo Ducasse, Audrey Arnal, Marion Vittecoq, Simon P Daoust, Beata Ujvari, Camille Jacqueline, Tazzio Tissot, Paul Ewald, Robert A Gatenby, Kayla C King, François Bonhomme, Jacques Brodeur, François Renaud, Eric Solary, Benjamin Roche, Frédéric Thomas

**Affiliations:** 1 MIVEGEC, UMR IRD/CNRS/UM 5290Montpellier Cedex 5, France; 2 CREEC, Université Montpellier 2Montpellier Cedex 5, France; 3 Centre de Recherche de la Tour du ValatArles, France; 4 Department of Biology, John Abbott CollegeSainte-Anne-de-Bellevue, QC, Canada; 5 Centre for Integrative Ecology, School of Life and Environmental Sciences, Deakin UniversityWaurn Ponds, Vic., Australia; 6 Department of Biology and the Program on Disease Evolution, University of LouisvilleLouisville, KY, USA; 7 Department of Radiology, H. Lee Moffitt Cancer Center & Research InstituteTampa, FL, USA; 8 Department of Zoology, University of OxfordOxford, UK; 9 ISEM Institut des sciences de l'évolution, Université Montpellier 2, CNRS/IRD/UM2 UMR 5554Montpellier Cedex, France; 10 Institut de Recherche en Biologie Végétale, Université de MontréalMontréal, QC, Canada; 11 INSERM U1009, Université Paris-Sud, Gustave RoussyVillejuif, France; 12 Unité mixte internationale de Modélisation Mathématique et Informatique des Systèmes Complexes (UMI IRD/UPMC UMMISCO)BondyCedex, France

**Keywords:** biomedicine, cancer, disease biology, evolutionary medicine

## Abstract

For an increasing number of biologists, cancer is viewed as a dynamic system governed by evolutionary and ecological principles. Throughout most of human history, cancer was an uncommon cause of death and it is generally accepted that common components of modern culture, including increased physiological stresses and caloric intake, favor cancer development. However, the precise mechanisms for this linkage are not well understood. Here, we examine the roles of ecological and physiological disturbances and resource availability on the emergence of cancer in multicellular organisms. We argue that proliferation of ‘profiteering phenotypes’ is often an emergent property of disturbed, resource-rich environments at all scales of biological organization. We review the evidence for this phenomenon, explore it within the context of malignancy, and discuss how this ecological framework may offer a theoretical background for novel strategies of cancer prevention. This work provides a compelling argument that the traditional separation between medicine and evolutionary ecology remains a fundamental limitation that needs to be overcome if complex processes, such as oncogenesis, are to be completely understood.

## Introduction

Cancer, a disease of multicellular organisms, probably developed almost immediately following the transition from unicellular to metazoan life, about one billion years ago (Merlo et al. 2006; Aktipis and Nesse 2013; Nunney 2013). The existence of multicellular organisms requires cooperation among cells so that the morphology and proliferation of each individual cell are controlled by instruction from the organism (Maynard-Smith and Szathmàry 1995). In other words, the Darwinian unit of selection is the whole organism and not individual cells. A major problem faced by any cooperative system (see West et al. 2007) is that they are vulnerable to exploitation by cheaters. Cheaters are individuals that have access to group benefits, but partially or completely withhold their fair share of contributions to those benefits (Buss 1987; Maynard-Smith and Szathmàry 1995; Michod 1999). Selfish traits emerge from natural selection acting at multiple levels as cheaters gain individual advantage over the cooperative group that they exploit. In multicellular organisms, cooperating somatic cells give up their own reproductive interests to better propagate their shared genetic material. In contrast, cancer cells develop a self-defined fitness function in which their proliferation is dependent solely on its fitness within the context of the local adaptive landscape. Conflict between multicellular tissues and single cancer cells is thus central to understanding the pathogenesis and evolution of cancer (Nunney 1999; Merlo et al. 2006; Greaves 2007; Pepper et al. 2009; Thomas et al. 2013). While cooperative systems can theoretically be ruined by cheaters that decrease group fitness, intraspecific cheating occurs at low frequencies in the wild (e.g., Gilbert et al. 2007 for microorganisms; Barron et al. 2001 and Hughes et al. 2008 for eusocial insects). This is largely because various mechanisms have evolved to prevent cheater individuals from exploiting/parasitizing the collective (Frank 1995; Gardner and West 2004). This is also the case with cancer, as initiation of tumors in multicellular organisms seems unavoidable while their progression to malignancy is most often prevented (Bissell and Hines 2011; Holly et al. 2013). For instance, most individuals (at least in humans) harbor precancerous lesions and *in situ* tumors in a variety of organs (e.g., prostate, lung, thyroid, breast, pancreas) (see for instance Rich 1935; Franssila and Harach 1985; Nielsen et al. 1987; Sakr et al. 1993; Folkman and Kalluri 2004; Manser et al. 2005; Bissell and Hines 2011), but they do not necessarily lead to the development of malignant cancers (Folkman and Kalluri 2004). Strong constraints on somatic evolution to suppress cancer have evolved along with multicellularity; individuals with unregulated cell division were at a selective disadvantage over those that were able to prevent uncontrolled cell proliferation (Casás-Selves and DeGregori 2011; Aktipis and Nesse 2013). This undoubtedly explains why complex organisms evolved many potent cancer suppression mechanisms (at least through the period of sexual maturity and reproduction, Campisi 2003). These include cell-intrinsic checks that prevent cells from becoming cancerous, to integral controls that suppress cancer by operating at the level of tissue organization (see Ewald 2009; Casás-Selves and DeGregori 2011). Considering the trillions of cells in the human body, the multitude of possible mutations that can or do occur and the ensuing genomic instability, we can conclude that our ability to restrain the aberrant growth and behavior of precancerous cells is extremely efficient (Bissell and Hines 2011).

Because of the deleterious effects of the unrestricted proliferation of cancer cells, cancers are analogous in many ways to the evolutionary and ecological dynamics of invading entities that harm cooperative and/or structured systems. Understanding the ecological contexts that promote or restrain proliferations in normally robust systems may provide insights into comprehending why some neoplasms develop into lethal tumors while others remain indolent for decades (see for instance Crespi and Summers 2005). The proliferation of cheaters is a common property of disturbed, resource-rich environments at all levels of biological organization (e.g., population, community, ecosystem, human society, eusocial insects, social ameba, and social bacteria). We review here the evidence for this phenomenon, provide detailed description of the underlying causes, and suggest a theoretical perspective/framework in relation to oncogenesis. Finally, we elaborate on how acknowledging the applicability of these principles to tumor formation could provide valuable insights into identifying common evolutionary routes for cancer dynamics and offer novel strategies for prevention.

## Resources, disturbances and proliferation of profiting phenotypes at different biological scales

### Ecosystems

The invasion of a new species into an established ecosystem is determined not only by the characteristics of the invaders, but also by the invasibility of the ecosystem itself, which depends on several biotic and abiotic characteristics (see Mack et al. 2000; Catford et al. 2012 for reviews). For plant communities, Davis et al. (2000) proposed a general hypothesis of invasibility based on fluctuating resource availability with communities becoming more susceptible to biological invasion whenever there is an increase in the amount of available resources. This theory rests on the simple assumption that an invading species can indeed proliferate only if it has access to resources and does not encounter intense competition from resident species. This assumption originates from the principle that competition intensity should be inversely correlated with the amount of unused resources (Davis et al. 1998). Several theoretical models and empirical studies support this hypothesis. For instance, increasing water supplies in soil indirectly enhances the susceptibility of herbaceous communities by invasive woody and herbaceous vegetation (Grime and Curtis 1976; Harrington 1991; Li and Wilson 1998; Davis et al. 1999). Conversely, imposed drought decreases the invasibility of the same communities (Davis et al. 1998). The role of ecological disturbances in Davies et al.'s theory is important but mostly interpreted through their effects on resource availability because in the context of plants, disturbances are likely to introduce additional resources into the community and/or lead to a decline in the global resource uptake due to mortality or debilitation of the resident species.

### Cooperative systems

Several nonmutually exclusive mechanisms have been shown to favor the evolution and maintenance of cooperation among individuals (West et al. 2007). In addition to reciprocal benefits, enforcement (e.g., punishment, policing, etc.) appears to be a key component in the maintenance of cooperative behaviors. Here, cooperation is encouraged through specific adaptations in the social environment that function to make defection costly. It is important to note that social enforcement mechanisms are dependent on specific environmental conditions to operate optimally; a disruption in the environmental homeostasis could lead to a reduction in the efficiency or even a breakdown of social enforcement. In the following sections, we will overview the impact of stochastic disturbances on social enforcement mechanisms at different biological scales and discuss how they may influence the spread of cheater phenotypes.

### Community

Cooperation between different species, defined here as mutually beneficial interspecific interactions, plays a central role in the functioning of all ecosystems (Ferriere et al. 2002). Indeed, every species on earth is involved directly or indirectly in one or several mutualistic associations (Bronstein et al. 2004), which are central to their survival and reproduction (e.g., pollination, seed dispersal, etc.) (Stachowicz 2001; Kremen et al. 2007). Such associations are dynamic at both ecological and evolutionary timescales; changes in biotic and abiotic conditions can lead to shifts from once-beneficial mutualistic exchanges to less beneficial or even detrimental antagonistic associations (i.e., cheating) (West et al. 2007).

It is becoming apparent that human impact on global ecosystems can rapidly alter the cost-benefit trade-offs associated with cooperation (Palmer et al. 2008), destabilizing existing mutualistic partnerships and promoting shifts toward antagonism (Johnson 2010). For example, repeated and prolonged drought episodes in Mediterranean forests have created environmental conditions that select against water-saving benefits conferred by leaf endophyte mutualists (Moricca and Ragazzi 2008). Beneficial endophytic leaf partners have been found to adopt growth patterns that allow them to aggressively colonize weakened, dry tree tissues, facilitating their ability to exploit hosts as water becomes limiting (Moricca and Ragazzi 2008). Another example is that of the impact of fertilizer use on the mutualistic associations between legumes and mycorrhizal fungi. Briefly, photosynthetic plants harness the solar energy of the sun to synthesize organic molecules from CO_2_, water, and minerals. Mycorrhizal symbiosis increases the fitness of plants living in mineral-deficient soils because fungi provide plants with access to limiting soil minerals, and in return, the plant provides the mycorrhiza with organic carbon. Mounting evidence suggests that fertilizer use may be detrimental to the persistence of plant–mycorrhizal mutualisms (Johnson 2010): Enrichment with fertilizers decreases the nutrient limitation that makes mycorrhizal mutualists beneficial and can lead host plants to severely decrease or cease resource allocation to their partners. This has been predicted to shift the competitive balance among mycorrhiza, favoring the evolution of more aggressive, antagonistic genotypes under increasingly high nutrient conditions (Thrall et al. 2006). These examples highlight that, even in systems where policing mechanisms are not needed for the maintenance of cooperative strategies, stochastic disturbances and increased resource availability favor noncooperative strategies.

### Social organization/interaction

As expressed by Nowak (2006), ‘humans are the champions of cooperation’, as cooperative behaviors have formed the bedrock of human societies throughout the ages. As we are intimately aware, modern societies are extremely dependent on the cooperation between individuals, cities, states, and countries to function properly. Interestingly, this also holds for hunter-gatherers, who typically exploit dense networks of exchange relations and practice sophisticated forms of food-sharing, cooperative hunting, and collective warfare (Fehr and Fischbacher 2004; Nowak 2006). In addition to the genetic, physiological, and psychological factors that are thought to be involved in the evolution of cooperative behaviors in humans (reviewed in Bowles and Gintis 2003; Fehr and Fischbacher 2004; Nowak 2006), social sanctions (laws, religious codes of conducted, cultural norms, etc.) and their enforcement mechanisms (e.g., social pressure, religious persecution, policing, etc.) also appear to be crucial for their maintenance in highly structured societies (Bowles and Gintis 2003; Fehr and Fischbacher 2004). This allows higher levels of cooperation to evolve and stabilize among unrelated individuals and in large groups (see Melis and Semmann 2010 for review).

Stochastic disturbances, such as natural disasters (e.g., earthquakes, volcanic eruptions, tsunamis, hurricanes, epidemics, floods) and human-caused catastrophes (e.g., toxic spills, nuclear accidents, plane crashes), have been shown to significantly disrupt social order (increased incidences of looting, physical abuse, divorce, etc.) most likely by reducing the efficacy of enforcement mechanisms (Nel and Righarts 2008), thus giving meaning to the old adage ‘when the cat is away, the mice come out to play’. For instance, a recent study, utilizing a comprehensive data set encompassing 187 international political entities from 1950 to the present, provided robust evidence that stochastic natural disasters significantly increased the risk of violent civic conflicting in the short term and medium term (Nel and Righarts 2008). Interestingly, the relationship between the number of climate-related natural disasters experienced and the risk of violent civil conflict is curvilinear, tracing an inverted U, peaking at rare (1–2) and frequent (>5) disturbances. Furthermore, these effects are magnified in countries that have intermediate to high levels of inequality, mixed political regimes, and sluggish economic growth (Nel and Righarts 2008).

### Eusocial organization/interaction

Social insects (e.g., ants, termites, bees, wasps) provide a classic example of extreme biological cooperation characterized by a well-developed reproductive division of labor between queens and workers within colonies. Although best known for cooperation, complex insect societies are also vulnerable to parasitic attack from reproductive workers. Indeed, while workers usually cannot mate nor lay fertilized eggs, in certain species, they have retained the capability of producing males from unfertilized eggs. This selfish reproduction among group members is called ‘social cancer’ and may lead to the death of the colony in interspecific context (Oldroyd 2002). The African honeybee *Apis mellifera scutellata* of South Africa provides a nice illustration of a lethal and highly contagious ‘social cancer’ due to self-replicating workers. Since 1990, a clone of *A.m. capensis* workers has been invading colonies of *A.m. scutellata* and parasitizing brood with their eggs, causing the host *A.m. scutellata* to raise yet more parasitizing workers (Martin et al. 2002). A cheater lineage has also been observed in the Japanese ant *Pristomyrmex punctatus* (Dobata and Tsuji 2009), a species characterized by asexual reproduction and lack of a division of labor. All females fulfill both reproduction and cooperative tasks in the colony. Cheaters lay more eggs and take little part in cooperative tasks. Greater availability of food is apparently a key variable explaining why cooperator phenotype adopts a cheater trajectory (Dobata and Tsuji 2009).

### Microorganisms

Although most evidence comes from laboratory conditions, it is now well established that some microorganisms exhibit cooperative, altruistic, and exploitative behaviors that are analogous to those observed in higher eukaryotes (Crespi 2001; Velicer 2003; Wingreen and Levi 2006). For instance, fruiting body construction in the myxobacteria and eukaryotic slime molds, biofilm and quorum-sensing systems in bacteria illustrate such cooperative interactions (See Crespi 2001 for review). In bacteria, the effects of resources and disturbances on cooperation are complex, depending on the spatial and temporal structure of the environment (MacLean and Gudelj 2006), and also because disturbance frequency and intensity may not have equivalent effects. It has been shown in the bacteria *Pseudomonas fluorescens* that intermediate disturbance frequencies favors the cooperative trait of biofilm formation (Brockhurst et al. 2007) because disturbances cause population bottlenecks, increasing relatedness among bacteria, thereby favoring cooperation. Interestingly, very high frequencies of disturbance cause population densities to fall below that required for cooperation to be beneficial, while very low frequencies of disturbance allow evolved cheats to accumulate. Several studies confirmed that resource supply is an important factor in the evolution of cooperation (Brockhurst et al. 2008), reducing the costs of cooperation. Therefore, the fitness of cooperators and by extension their frequency within the population increases with increasing resource supply. However, further studies would be necessary before generalizations can be made on the positive effects of resource abundance on cooperation in bacteria.

The *Dictyostelia* or social amebas represent a unique form of multicellularity that has the particularity to be conditional. Indeed, under nutrient-rich habitats, these soil organisms are unicellular, but when food supply is depleted, cellular agglomerates of up to a million of amebas are formed to generate a motile structure, called the ‘slug’, which responds to chemical gradients and can migrate to light and warmth, that is soil's top layer (Schaap 2007). Then, the sequence ends in a process called culmination and the slug forms the fruiting body in which a proportion of cells are sacrificed to build the stalk and the remainder differentiate into resilient dormant spores (Schaap 2011). The transient multicellularity in amebas is a response to starvation, which indirectly suggests that selfish forms are favored when resources are abundant.

## Cancer

### Stressors, caloric intake, and cancer

Anecdotal evidence of a link between stress and disease progression is quite common in the developed world (Glaser and Kiecolt-Glaser 2005; Chida et al. 2008). More recently, mounting epidemiological and clinical data [e.g., twofold increase in breast cancer risk in women following a divorce, separation or death of a spouse (Lillberg et al. 2003), increased risk of lung cancer in men associated with job instability and death of a spouse (Horne and Picard 1979)], in concert with studies elucidating the mechanisms involved in stress-initiated and stress-enhanced cancers have provided empirical evidence for a link between stress and cancer emergence (Glaser and Kiecolt-Glaser 2005; Antoni et al. 2006; Kemeny and Schedlowski 2007).

Once initiated via a stressor, the dynamics of cancer progression can be additionally affected by diet (i.e., caloric intake; Hursting et al. 2009). High-fat and sugar diets not only contribute to cardiovascular diseases and diabetes, but also significantly exacerbate cancer proliferation (Hursting et al. 2009). Due to growing clinical evidence and recent experimental studies identifying potential biological signaling pathways involved, calorie restriction (CR) is being heralded as the most potent, broadly acting dietary regimen for suppressing the carcinogenesis process (Hursting et al. 2009). Although the proximate mechanisms involved in stressor initiation and resource enhancement of cancer are relatively well understood, the evolutionary underpinnings remain largely unexplored. Investigating the joint role of CR in aging and oncogenesis could provide some evolutionary explanations. As CR appears to slow aging processes in a range of animal species (Weindruch and Walford 1988; Chapman and Partridge 1996; Houthoofd et al. 2002), it has been proposed that the response to limited food supply could represent a generalized evolutionary adaptation, potentially a strategy to cope with periods of famine (Kirkwood and Shanley 2005). CR organisms increase investment in cellular maintenance functions over reproduction, which may increase survival with a concomitant reduced intrinsic rate of senescence and hence enhance fitness once nutritional resources are restored (Kirkwood and Shanley 2005). CR contributes to lifespan extension by affecting the same metabolic and physiological pathways (i.e., growth factors, anabolic hormones, inflammatory cytokines, and oxidative stress markers) involved in oncogenesis (Longo and Fontana 2010). Therefore, we propose that the two processes, aging and oncogenesis, could have joint evolutionary histories, and the role of CR in controlling malignant formation could be an evolutionary spin-off of the adaptation to food restriction.

## Theoretical exploration

To illustrate our verbal arguments, we developed a simple mathematical model to examine how sporadic disturbances, on immune system efficiency and resource supply, can yield a significant increase of cancerous cell accumulation. We used the following framework:

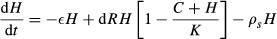


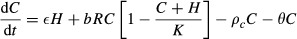


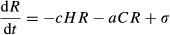


Within this mathematical framework, we considered two populations of cells: healthy (H) and cancerous (C). Each population of cells dies at a rate *ρ*, which differs between healthy and cancerous phenotypes, and uses the resources consumed to allow the creation of new cells at a rate *d* and *b,* respectively, while resources (R) are consumed by healthy and cancerous cells at rates *c* and *a,* respectively. Resources are added constantly through time at rate *σ*. We also assumed that the total number of cells cannot exceed a carrying capacity *K*, inducing then a competition between healthy and cancerous cells.

Cancerous cells arise at a rate *ɛ* from the healthy cell population. However, population size of cancerous cells needs to reach some threshold to start an efficient proliferation. Indeed, a cancerous cell alone cannot propagate quickly and needs different factors (such as angiogenesis, see Hanahan and Weinberg 2011) to replicate. To mimic such fundamental requirement of tumor growth, we assumed that cancerous cells start consuming resources only when their population size reaches a given threshold. We thus assumed that *b* rate is driven by the following Gompertz function (a classic sigmoid relationship):

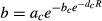


where *a*_*c*_, *b*_*c*_, and *d*_*c*_ are constant parameters shaping this threshold. Finally, cancerous cells are eliminated by immune system with rate ***θ***.

**Figure 1:**
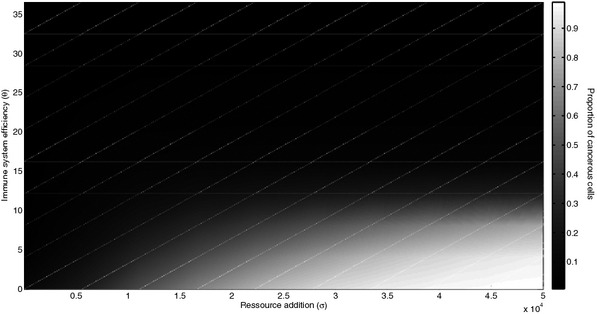
Influence of immune system efficiency and resource supply on the appearance of cancerous cells. A decrease in *y*-axis represents a permanent diminution of immune system efficiency while an increase in *x*-axis represents a permanent addition in resources supply. Colors represent proportion of cancerous cells following a black to white gradient. Parameters: *ε* = 365/1000 cell^−1^ year^−1^, K = 10^3^, *ρ*_*s*_ = 365/40 cell^−1^ year^−1^, *ρ*_*c*_ = 365/15 cell^−1^ year^−1^, a = 365 cell^−1^ year^−1^, c = 365/10 cell^−1^ year^−1^, d = 365/30 cell^−1^ year^−1^, a_c_ = 365/8 cell^−1^ year^−1^, b_c_ = 10^5^, d_c_ = 15.

We first analyzed the influence of the combination of immune system efficiency and resource supply on the frequency of cancerous cells at the equilibrium without any sporadic disturbances. Figure1 shows that both a decrease in immune system efficiency and an increase in resource supply are needed to reach high levels of cancerous cells.

**Figure 2:**
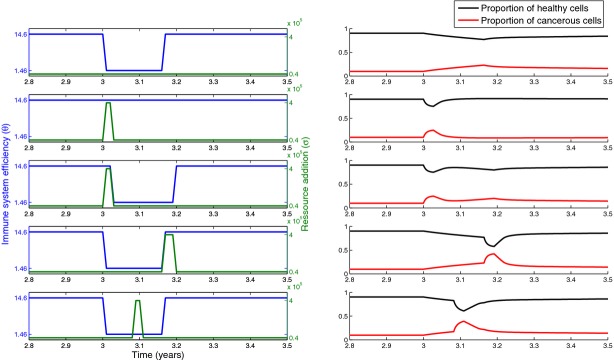
Profiles of disturbances (left) and consequences for cancerous cell population dynamics (right). (First line) Disturbance on immune system efficiency only. (Second line) Disturbance on resource supply only. (Third line) Disturbance on resource supply occurs before disturbance on immune system efficiency. (Fourth line) Disturbance on resource supply occurs after disturbance on immune system efficiency. (Fifth line) Disturbance on resource supply occurs during disturbance on immune system efficiency. *θ* = 365/25 cell^−1^ year^−1^, *σ* = 4.10^4^ year^−1^, other parameters are identical to those in Fig.1.

Then, we tested the role of sporadic disturbances. Starting with an immune system efficiency and a level of resource supply that do not allow a high level of cancerous cells, we added sporadic disturbances on immune system efficiency (duration of 2 months, to mimic immunosuppression that could be observed during winter time) and resource input (duration of 10 days, to mimic a too rich diet for a special event like festive meals). Figure2 shows that a burst of resources seems to play a larger role on the appearance of cancerous cells than immune system. This pattern is mainly driven by the higher production rate of cancerous cells compared with healthy ones, which allows cancerous cells to be more competitive and partly outcompete healthy cells temporarily following an additional resource supply.

These simulations also suggest that the timing of disturbances is an important predictor of sporadic accumulation of cancerous cells. The worst timing follows a disturbance on resource supply occurring during or after disturbance on immune system. As disturbance on resource supply has a larger impact than disturbance on immune system efficiency in our theoretical framework, immunosuppression allows an increase of cancerous cells frequency that is then amplified by resource input. Nevertheless, because quantifying strength of immunosuppression or level of additional resource supply deserves a full study, we cannot conclude which process is the most important.

This initial theoretical approach aims at showing that each type of disturbance may have a different outcome on cancerous cell population dynamics. It also underlines the strong influence of the timing of such disturbances. The simplicity of this model does not allow studying the influence of these sporadic disturbances may have in the long term because more sophisticated processes should be then considered. For instance, reaching 50% of cancerous cells among the whole cell population (as our simulations show), even temporarily, definitely increases the probability of metastasis and jeopardizes prognosis of the individual. Indeed, such proportion is expected to break down the homeostasis of the organism considered, with important consequences on the individual health that we do not address here. While addressing these complex mechanisms goes over the edges of the current study, we believe that such theoretical framework should be extended and analyzed deeply.

## Implications for cancer prevention

The proliferation of profiteering phenotypes is often an emergent property of disturbed, resource-rich environments relevant at all scales of biological organization. Whether or not carcinogenesis, which can be viewed as the proliferation of profiteering/cheating cells, is also governed by this principle is a legitimate question. We argue that instead of being a distinct phenomenon, cancer is a particular manifestation of a quasi-universal ecological process that is the proliferation of profiteering phenotypes in disturbed systems with unused resources. Several studies have compared the metastatic cascade to biological invasions by exotic species (e.g., Gatenby et al. 2009; Chen and Pianta 2011). While this comparison is acceptable for several ecological and evolutionary reasons, we would like to extend the theory to the development of primary tumors.

Our concept aligns closely with the general theory of invasibility proposed for plant communities as described above (Davis et al. 2000; Mack et al. 2000). However, there is one fundamental difference: while disturbances directly act on the level of available resources in plant communities, the primary detrimental effect of disturbances in carcinogenesis is through the disruption of natural barriers against undesirable invasions (notably policing, e.g., immune system). By analogy with this ecological literature, two key conditions must be met for an invasive cancer to develop: (i) available resources for seeds to germinate, and (ii) damaged protective barriers unable to inhibit the proliferation/invasion. Analyzing in more detail how we could potentially act on these two conditions and/or their interactions may offer a theoretical background for novel strategies of cancer prevention.

### Presence of unused resources

Because weight gain leading to overweight occurs when energy intake chronically exceeds energy expenditure (Romieu et al. 1988), ponderosity (body weight relative to height) can be interpreted as a surrogate of unused resources. Our ancestors evolved from a nutritional landscape very different from today. This is especially true in westernized societies in which the diet is characterized by large amounts of high-calorie and high-fat food. Eating in the past also required high-energy investment which made it impossible for most people to accumulate much surplus as fat (Eaton and Konner 1985). This mismatch between ancestral conditions and current lifestyles results in several health problems, such as obesity, hypertension, diabetes, and cancers (Nesse et al. 2012). Numerous studies have successfully linked calorie intake and cancer and identified several of the mechanisms involved (e.g., hormones and growth factors, insulin, IGF-1, leptin, adiponectin, steroid hormones, inflammation, sirtuins; see Hursting et al. 2009 for a review of the past 30 years of CR research). Calorie surplus leads to the presence of available resources and is therefore likely to favor invasive cancers that rely on the rapid cellular growth which itself depends on an increase in supply of energy and of substrates for the biosynthesis of all the macromolecules required to build new cells. In accordance with this hypothesis, the incidence of cancer is increasing in developing countries owing to changes in lifestyle and diet that occur with economic development (consumption of highly refined foods, sugars and/or saturated fat; Prentice and Sheppard 1990; Bergström et al. 2001; Bianchini et al. 2002; Rastogi et al. 2004). Cancer progression also likely depends on the energy balance (nutritional excess/lack of activity) and composition of diet rather than simple calorie intake (Holly et al. 2013). In accordance with the idea that the presence of unused resources favors the ability of seeds to germinate, not the quantity *per se*, an interesting parallel could be made to countries where rapidly changing lifestyles resulted in diet having a more significant effect on the progression of cancers to clinical stages than on the initiation of latent cancers (Shiraishi et al. 1994; Holly et al. 2013). Theoretically, natural selection could eventually fix these oncogenic disorders (as well as other calorie associated conditions), but hundreds or thousands of generations would be required, and the selection pressure would only have influence on cancers occurring through the period of sexual maturity and reproduction. An efficient solution to reduce the risks of cancer progression and metastasis would be to adopt a low-calorie diet. During high-caloric restriction, to ensure cellular survival, autophagy, a catabolic mechanism, degrades unnecessary or dysfunctional cellular components. The contributing role of autophagy in the context of CR-induced health benefits has been recently unraveled (Pallauf and Rimbach 2013). Genetic inhibition of autophagy induces degenerative changes in mammalian tissues that resemble those associated with aging, and normal and pathological aging are often associated with a reduced autophagic potential. Pharmacological or genetic manipulations that increase lifespan in model organisms often stimulate autophagy, and its inhibition compromises the longevity-promoting effects of caloric restriction, activation of the deacetylase sirtuin 1, inhibition of insulin/insulin growth factor signaling, or the administration of rapamycin, resveratrol, or spermidine (Steeves et al. 2010; Rubinsztein et al. 2011). However, this solution is difficult to accept for many people, presumably because we also have been selected to appreciate sweet and fatty foods (Nesse et al. 2012). Facing the dramatic increase of overweight and obesity in many countries, enhancing educational alimentation remains crucial to fight bad food habits. Alternatively, the identification of drugs that could boost exercise endurance (Woldt et al. 2013) or either complement or even reproduce the anticancer effects of CR without drastic changes in diet and lifestyle (i.e., CR mimics) is a goal for many pharmaceutical companies (Hursting et al. 2009). Elucidating the mechanisms underlying the anticancer effects of CR and exploiting this mechanistic information to target calorie restriction-responsive pathways through combinations of dietary and pharmacologic approaches should permit in the future the development of effective cancer prevention strategies in humans.

The link between diet and cancer is not only quantitative, but dietary constituents have been shown to qualitatively modulate the complex multistage carcinogenesis process at the initiation, promotion, and progression phases of neoplasia (Milner et al. 2001; Go et al. 2003). This occurs because both essential nutrients and nonessential bioactive food components can alter many of the pathways of cancer, including apoptosis, cell cycle control, differentiation, inflammation, angiogenesis, DNA repair, and carcinogen metabolism (see Ross 2003, 2010 for review). This can also occur indirectly, through the modification of the microbiome that is increasingly recognized for its important functions in health and diseases, including cancer (Kinross and Darzi 2011; Cho and Blaser 2012; Schwabe and Jobin 2013). In humans, microbes, both commensal and pathogenic, are critical regulators of the host immune system and, ultimately, of inflammation. Consequently, microbes have the potential power to influence tumor progression as well, through a wide variety of routes, including chronic activation of inflammation, alteration of tumor microenvironment, induction of genotoxic responses and metabolism. The impact of gut microbiota in eliciting innate and adaptive immune responses beneficial for the host in the context of effective therapies against cancer has been highlighted recently: the anticancer efficacy of alkylating agents (such as cyclophosphamide) and platinum salts (oxaliplatin, cisplatin) is compromised in germ-free mice or animals treated with antibiotics (Viaud et al. 2013). Gut microbiota could also be involved in the link between obesity and increased cancer risk through overproduction of a DNA-damaging bile acid (Ohtani et al. 2014). It is relevant in this context to recall that the shift to modern diet habits is also characterized by significant qualitative changes (e.g., our ancestors evolved on a diet that included daily intake of fiber from a diversity of sources; Leach 2007). Improving our knowledge on the qualitative aspects of our ancestral bowels is important because they also conditioned our current nutritional parameters and physiological responses. The requirement range of particular nutrients may also be contingent upon the functionality of the cell and organism (Go et al. 2003). Certain nutrients may also be harmful in supernormal doses. Determining whether qualitative changes in our diets contribute to the presence of unused resources and/or are equivalent to disturbances for our body is thus unclear at the moment. In general, despite considerable progress in our understanding of the relationship between diet and cancer much remains to be revealed with respect to the relationship between cancer risk and our exact dietary requirements, constituent absorption and metabolism (Schoenfeld and Ioannidis 2013).

The links between cancer and diet can be initiated early in life, commencing from embryogenesis and fetal development, through early childhood when complicated interactions of multiple environmental factors, including diet, influence the developmental trajectories and physiology resulting in subsequent increase in cancer risk later in life (Frankel et al. 1998). For example, *in utero* exposure to certain so-called epigenetic diets that affect key tumor-related gene expression through epigenetic regulation may lead to reprogramming of primary epigenetic profiles of the fetal genome, resulting in different susceptibility to diseases, including cancer (Li et al. 2014). For example, genistein in soybean products, sulforaphane in cruciferous vegetables, and epigallocatechin-3-gallate (EGCG) in green tea have been associated with a lower risk of developing several common cancers and are considered as dietary epigenetic modulators. Dietary epigenetic intervention could provide a cost-effective transgenerational human disease control, and the prenatal and/or postnatal dietary administration of epigenetic supplements (with chemopreventive potential) could lead to early cancer prevention (Li et al. 2014).

### Avoiding disturbances to prevent cancer: an underestimated solution?

While loss of homeostasis is traditionally seen as a key initial step on the route to cancer development (Hanahan and Weinberg 2000, 2011), much remains to be performed to fully understand disturbances that weaken homeostasis at a level sufficient to initiate an invasive cancer. Defining disturbances in the context of cancer risk is challenging because it, firstly, concerns several levels of biological organizations, ranging from cells, tissues, organs, and individual. Secondly, the amplitude and timescale of disturbances, punctual or chronic episodes resulting from evolutionary mismatches between ancestral and modern life styles, can also be essential. At the moment, we mostly possess a qualitative knowledge, namely a list of variables/situations that favor cancer initiation by presumably acting as disturbances (depression, infections, lack of sleep, stress…), but we miss quantitative information on them (e.g., intensity, frequency, length of exposure) in the context of cancer risk. For instance, we know that immunosuppression, whatever its origin, can favor cancer progression, but we do not know precisely the shape of the relationship between the lengths of time people must stay in an immunosuppressive state for an invasive cancer to progress until problematic stages. Undoubtedly, this relationship also depends on the complex interaction of individual parameters such as the age, psychology, life style, and genetic background. Furthermore, numerous cancers have an infectious causation (Zur Hausen 2009), and typically oncogenic pathogens induce disturbances at cellular level that favor cancerous transformation because they alter natural barriers to tumorigenesis (see Ewald 2009).

Only once precise quantitative data will be obtained on the links between disturbances and invasive cancer initiation, will it be possible to formulate realistic predictions on cancer probabilities in relationship with personal characteristics. It is increasingly acknowledged that accompanying actions such as psychological support and/or pain avoidance reduce oncogenic progression in cancerous patients (Chida et al. 2008). This is in accordance with the idea that disturbances have an exacerbating effect on cancerous progression. What we suggest here is that any actions that reduce the level of disturbances prior to the initiation of oncogenic progression should be developed because they are expected to prevent oncogenesis. This is indirectly supported by several studies linking different lifestyles and psychological profiles to cancer risk (Kreitler et al. 2013).

The elusive nature of malignancy initiations, and its progression until a threshold, above which it becomes problematic to stop, arises from the fact that it depends upon conditions that occur intermittently. One important corollary of this is that susceptibility to invasion by cancerous cells is not a static or permanent attribute, but a condition that fluctuates over time, with changes from year to year and even within a given year, as the amount of unused resources and of disturbances fluctuates. Establishing causative correlations between particular disturbing events during the life and subsequent cancer risk, sometimes years later, is undoubtedly challenging and should be studied first on animal models where genetic and ecological parameters can be experimentally controlled. This research should ultimately contribute to the development and efficiency of preventive behaviors by providing a clearer objective necessary for obtaining efficient protections against cancer. Finally, such knowledge should permit to develop concrete therapeutic preventive strategies. Being able to identify the episodic events during which cancer initiation and subsequent progression are likely to occur is indeed crucial, because it could lead to the development of novel preventive strategies that focus specifically on those critical periods.

Broadly, a first direction could rely on treatments whose basic principle is to prevent people from being in a disturbed state likely to promote carcinogenesis. This strategy has been tested in infectious diseases; for instance, antibiotics are used in fragile patients with a viral infection to prevent subsequent bacterial infections that could develop in the immunosuppressed context induced or majored by the virus. A second possibility would be to propose treatments specifically against cancer initiation when people cannot avoid crossing a risky period. For example, a specific treatment against cancer cells can be proposed when people experience depressive disorders. Once again, this strategy is successfully applied in some infectious diseases, for example where antimalarial medication is recommended to people visiting a malaria-endemic region.

### Environmental disturbance, infection and cancer

Oncogenic parasites, as a rule, cause cancer in only a small percentage of infected individuals. This finding and the high frequency of mutations in pathogen-induced cancers have led to the conclusion that pathogen-induced oncogenesis almost always requires mutations in hosts (Zur Hausen 2010). The corollary of this conclusion is that increased exposure to mutagens will increase the frequency of pathogen-induced cancers. Environmental disturbances that increase exposure to mutagens therefore can be expected to increase pathogen-induced cancers.

Although there is little evidence to test this prediction in wildlife, the available information is consistent with it. Chemical pollution and fibropapilloma-associated turtle herpesvirus have been associated with sea turtle fibropapillomatosis, and levels of polychlorinated biphenyls are elevated in the blubber of genital carcinoma of sea lions (Herbst and Klein 1995; Foley et al. 2005; Ylitalo et al. 2005; McAloose and Newton 2009). Depending on the species under consideration, environmental disturbances that increase exposure to mutagens may enrich or deplete resources. Correlations between environmental richness and cancers may therefore need to consider the possibility that infectious agents and mutagens contribute jointly to oncogenesis.

By altering population densities of oncogenic parasites, ecological disturbances can alter the rate at which they cause cancer. Dam building, for example, has increased the populations of alternate hosts of human parasites that play a role in cancer. Increases in snail populations increase rates of schistosome infection (Steinmann et al. 2006) and may thus increase rates of bladder cancer. Similarly, increases in mosquito density may increase malarial infections (Keiser et al. 2005) and thereby increase rates of Burkitt's lymphoma. As infectious agents of wildlife cancer are discovered, similar effects of human disturbances on cancer in wildlife through influences on the prevalence of infection will need to be investigated.

Viruses are particularly likely to be oncogenic agents in wildlife populations. As intracellular parasites, they often replicate their genomes by stimulating host cells to proliferate. This manipulation is accomplished by abrogating critical barriers to cancer, such as cell cycle arrest, apoptosis, and the maximum number divisions a cell can undergo (Ewald 2009; Ewald and Swain Ewald 2012). Viruses that have been associated with cancer in wildlife generally belong to viral groups that cause human cancers. In the summary of wildlife cancers associated viruses provided by McAloose and Newton (2009), for example, most of the implicated viruses accord with this generalization (Table1). The human viruses in these groups are generally transmitted by sexual contact and saliva, and sometimes by milk and needle-borne blood (Table1). Because these modes of transmission occur at infrequent intervals, they favor long-term persistence within individual hosts (Ewald and Swain Ewald 2013). A major mechanism by which such persistence is possible involves compromising the barriers to cancer mentioned above (Ewald and Swain Ewald 2012).

**Table 1:** Viruses associated with cancer in wildlife

Virus associated with cancer in nonhuman host	Hosts	Taxonomic group of viruses	Human cancer-associated viruses in same taxonomic group*	
			Virus	Transmission
Various retroviruses	Walleye pike (*Sander vitreus*); Atlantic salmon (*Salmo salar*); Attwater's prairie chicken (*Tympanuchus cupido attwateri*)	Retroviridae	Human T-lymphotropic virus-1 (o)	Sex, milk, blood
Woodchuck hepatitis virus	Woodchuck (*Marmota monax*)	Hepadnaviridae	Hepatitis B virus (o)	Sex, blood
Otarine herpesvirus-1	California sea lion (*Zalophus californianus*)	Gamma herpesvirus	Epstein-Barr virus (o); Kaposi Sarcoma-associated herpesvirus (o)	Sex & saliva
Fibropapilloma-associated turtle herpesvirus	Sea turtle (*Chelonia mydas*)	Alpha herpesvirus	Human herpes simplex virus-2(a)	Sex
Bandicoot papillomatosis Carcinomatosis virus-1	Western barred bandicoots (*Perameles bougainville*)	Papillomavirus–polyomavirus mosaic	Human papillomavirus (o) Merkel cell polyomavirus (o)	Sexual contact probably saliva
Rana virus-1	Leopard frog (*Rana pipiens*)	Iridoviridae	None	

* (o) = oncogenic; (a) = cancer-associated and possibly oncogenic.

Although the transmission routes of viruses that are associated with cancer in wildlife are not well understood, these considerations regarding selection for persistence within hosts and the routes of transmission in humans suggest that oncogenic viruses in wildlife may be disproportionately transmitted by sexual contact, saliva, and milk. These modes of transmission often lead to geographic discontinuities of viruses or viral subtypes in human populations (e.g., human T-lymphotropic viruses) and presumably would do so in wildlife populations. If ecological disturbances allow geographically separated populations to come into contact, increases in infectious cancers would be expected in the newly unexposed populations. Spread to populations of different species is also possible because oncogenic effects of viruses in related host species have been noted, for example, in the reticuloendothelial cancer of galliformes, and the fibropapillomas of turtles (McAloose and Newton 2009).

Although the spread of oncogenic viruses among different populations of a given species in response to environmental disturbance has not been studied in wildlife, environmental disturbance has been implicated in the spread of human viruses that directly or indirectly contribute to human cancer. Oncogenic variants of the human papillomavirus have increased in association with conditions of warfare (Grce et al. 1996). Hepatitis C virus, which is a cause of human liver cancer, spread globally in response to new opportunities for blood-borne transmission (Markov et al. 2009). The human immunodeficiency virus, which contributes to cancer indirectly through immunosuppression, spread in humans during the early stages of the AIDS pandemic in response to changes that affected movements of human populations (Ewald 1994; Faria et al. 2014).

## Concluding remarks

**Figure 3:**
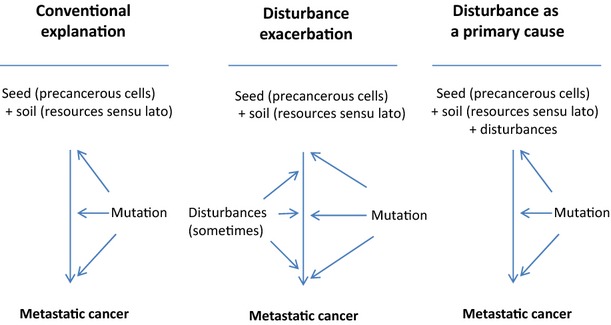
(A) The standard seed-soil hypothesis as an explanation for oncogenesis, (B) When the first associations between disturbance and cancer began surfacing they were accommodated by fitting then into the seed-soil paradigm, and (C) Disturbances play a more direct role because they compromise protective barriers to oncogenesis.

The traditional seed-soil hypothesis (Paget 1896) stipulates that, for metastatic cancers to start, the soil is as important as the seed. However, in the context of primary tumor, evidence indicates that ‘seeds’ are virtually everywhere in the body with aging and, at least in western lifestyle where caloric intake is high (true also in poor countries in which exposure to toxics and oncogenic pathogens is high), the ‘soil’ basically provides constantly favorable conditions for seeds—cancer progenitor cells—to germinate. From an ecological perspective, we argue here that disturbances constitute another key interacting parameter to include into the reasoning. The role of disturbances in carcinogenesis is well documented and generally accepted, but probably underestimated as a primary cause (Fig.3, case B versus case C). The distinction is crucial because preclusion of a primary cause prevents the disease, while inhibition of a secondary cause only reduces the frequency or severity of disease but does not prevent the disease itself. Ewald (2009) argued that we strongly underestimate the role of infections as cofactors of cancer initiation. We further argue here that we not only underestimate the role of disturbances and pathogens as cancer initiators, but also the role of additional factors, such as available resources and behavioral traits in potentially destabilizing homeostasis. As discussed above, it is challenging to act on both the seed presence and the soil suitability, but all the possibilities have not been sufficiently explored concerning the avoidance of disturbances. There is undoubtedly a very large range of disturbing effects, and causation is most of the time difficult to establish because time between cancer initiation and its detection is usually long. More ongoing research, especially in a quantitative context, should be performed to understand and potentially identify the range of disturbances that compromise natural barriers to cancer. Then, developing tools for monitoring homeostasis at all the different relevant scales should permit the identification of actual periods of the life that are the most at risks of the initiation of invasive cancers. From this knowledge, novel preventive strategies could be developed.
